# Can haematological changes constitute a surrogate diagnostic parameter to detect schistosomiasis in migrants and travellers? - A retrospective analysis

**DOI:** 10.1016/j.nmni.2023.101136

**Published:** 2023-04-27

**Authors:** Jenny L. Schnyder, Federico Gobbi, Mirjam Schunk, Andreas Lindner, Fernando Salvador, Alexandre Duvignaud, Marta Arsuaga Vicente, Jean Claude Dejon Agobé, Paolo Cattaneo, Giulia Bertoli, Camilla Rothe, Mia Wintel, Diana Pou, Denis Malvy, Ayola Akim Adegnika, Hanna K. De Jong, Martin P. Grobusch

**Affiliations:** a Centre for Tropical Medicine and Travel Medicine, Department of Infectious Diseases, Amsterdam University Medical Centers, Location AMC, Amsterdam Infection & Immunity, Amsterdam Public Health, University of Amsterdam, Amsterdam, the Netherlands; b Department of Infectious/Tropical Diseases and Microbiology, IRCCS Sacro Cuore-Don Calabria, Negrar di Valpolicella, Verona, Italy; c Division of Infectious Diseases and Tropical Medicine, LMU Hospital Centre, Munich, Germany; d Charité – Universitätsmedizin Berlin, Freie Universität Berlin and Humboldt-Universität zu Berlin, Institute of Tropical Medicine and International Health, Berlin, Germany; e International Health Unit Vall d’Hebron-Drassanes, Infectious Diseases Department, Vall d’Hebron University Hospital, PROSICS Barcelona, Barcelona, Spain; f Centro de Investigación Biomédica en Red de Enfermedades Infecciosas (CIBERINFEC), Instituto de Salud Carlos III, Madrid, Spain; g Department of Infectious Diseases and Tropical Medicine, Hôpital Pellegrin - CHU de Bordeaux, Bordeaux, France; h Imported Diseases and International Health Referral Unit, Hospital Universitario La Paz- Carlos III, Madrid, Spain; i Centre de Recherches Médicales en Lambaréné (CERMEL), Lambaréné, Gabon; j Institute of Tropical Medicine, University of Tübingen, German Centre for Infection Research, Tübingen, Germany; k Masanga Medical Research Unit (MMRU), Masanga, Sierra Leone; l Institute of Infectious Diseases and Molecular Medicine (IDM), University of Cape Town, Cape Town, South Africa

**Keywords:** Schistosomiasis, Haematology, Full blood count, Returned travellers, Travel, Migrants, *S. mansoni*, *S. haematobium*

## Abstract

**Background:**

Earlier studies found characteristic haematological changes in African patients with active schistosomiasis. If consistently present, full blood counts (FBC) may be helpful to diagnose schistosomiasis also in migrants and returning travellers.

**Methods:**

A retrospective patient record review was conducted on data from seven European travel clinics, comparing FBC of *Schistosoma* egg-positive travellers and migrants to reference values. Sub-analyses were performed for children, returned travellers, migrants and different *Schistosoma* species.

**Results:**

Data analysis included 382 subjects (median age 21.0 years [range 2–73]). In returned travellers, decreases in means of haemoglobin particularly in females (β = −0.82 g/dL, *p* = 0.005), MCV (β = −1.6 fL, *p* = 0.009), basophils, neutrophils, lymphocytes and monocytes (β = −0.07, *p* < 0.001; −0.57, *p* = 0.012; −0.57, *p* < 0.001 and −0.13 10^3^/μL, *p* < 0.001, respectively) were observed. As expected, eosinophils were increased (β = +0.45 10^3^/μL, p < 0.001). In migrants, a similar FBC profile was observed, yet thrombocytes and leukocytes were significantly lower in migrants (β = −48 10^3^/μL *p* < 0.001 and β = −2.35 10^3^/μL, *p* < 0.001, respectively).

**Conclusions:**

Active egg-producing *Schistosoma* infections are associated with haematological alterations in returned travellers and migrants. However, these differences are discrete and seem to vary among disease stage and *Schistosoma* species. Therefore, the FBC is unsuitable as a surrogate diagnostic parameter to detect schistosomiasis.

## Highlights

• Active schistosomiasis is associated with haematological changes in travellers and migrants.
• These differences seem to vary among disease stages and *Schistosoma* species.
• Full blood count (FBC) differences were discrete and inhomogeneous, hence unfit to develop a scoring system.
• The FBC seems unsuitable as a reliable surrogate diagnostic parameter to detect schistosomiasis.

## 1 Introduction

Schistosomiasis is a parasitic disease caused by blood flukes of the genus *Schistosoma*. It constitutes a major health problem in the tropics and subtropics. Transmission is reported from 78 countries, and approximately 240 million people are infected and require treatment, with over 90% of them living in sub-Saharan Africa [1]. *Schistosoma haematobium* is one of the major *Schistosoma* species and causes urogenital schistosomiasis characterised by haematuria and severe complications (hydronephrosis, renal failure, squamous cell carcinoma), whilst other *Schistosoma* species including *S. mansoni*, *S. japonicum*, *S. intercalatum* and *S. mekongi* mostly cause intestinal and hepatosplenic disease (bloody diarrhoea, portal hypertension and gastrointestinal bleeding). Symptoms are caused by inflammatory responses to schistosome eggs that are produced during the active infection stage by adult worms residing in blood vessels, and are subsequently excreted in urine or stool. To facilitate their extrusion from the blood vessel to the lumen of the urinary bladder or intestine, schistosome eggs actively secrete antigenic glycoproteins. These antigens, however, also induce formation of granulomas around the eggs trapping them in the surrounding tissue. This causes organ-specific complaints, such as haematuria in urogenital schistosomiasis or abdominal pain in intestinal schistosomiasis [2].

Several studies have been conducted on African individuals (mostly schoolchildren) living in endemic areas with active egg producing *S. haematobium* and *S. mansoni* infections, yielding characteristic haematological alterations compared to those not infected (see Supplementary Tables 1 and 2 for a literature overview) [[3], [4], [5], [6], [7], [8], [9], [10], [11], [12]]. These full blood count (FBC) characteristics slightly varied between *Schistosoma* species. In both populations infected with *S. haematobium* [[3], [4], [5], [6], [7]] and *S. mansoni* [[7], [8], [9],^11^,^12^], respectively, decreases in haemoglobin (Hb) and/or haematocrit were found (Supplementary Tables 3 and 4). This could be due to direct blood loss by the extrusion of eggs and/or consumption of erythrocytes by adult schistosomes [13]. Dejon Agobé et al., on the other hand, found that the decrease in Hb and haematocrit was not significant anymore, once adjusted for other soil-transmitted helminthic infections, *Plasmodium falciparum* co-infection, and use of praziquantel in the past six months. That notwithstanding, decreases in mean corpuscular volume (MCV) and mean corpuscular haemoglobin (MCH) remained statistically significant [3].

In addition, platelet counts were found to be higher in schoolchildren infected with *S. haematobium* [3,5], whilst results of studies on *S. mansoni* infected people varied [7,9,10,12,14]. An experiment in rats showed that thrombocytes acquire killing properties against schistosomes, that are highest 6–8 weeks after infection with *S. mansoni*. This is accompanied by an up to more than three-time increase in thrombocytes that peaks 42 days after infection [15]. On the long term, however, an infection with *S. mansoni* can progress to hepatosplenic schistosomiasis, which is associated with low platelet counts, due to the effects of hypersplenism and portal hypertension [10,[16], [17], [18]]. This might explain the decrease in thrombocytes found in a study on Ethiopians with egg-producing *S. mansoni* infections, as only the individuals from a community were included that attended the local hospital, which are often patients with more advanced disease [10].

Furthermore, studies on Gabonese and Sudanese schoolchildren found a significant increase in the mean counts of all differential leukocyte types [3,4]. As well, in Ghanaian schoolchildren, a small but statistically non-significant trend was found [5]. The high leukocyte count might be due to stimulation of the host immune system against schistosomes and/or their eggs. On the contrary, studies on *S. mansoni* infected people, observed a decrease in leukocyte count [8,9,11,12], with lower leukocyte counts associated with a higher intensity of infection, quantified by the amounts of eggs detected in the stool [11]. The above-described decrease in Hb and increases in thrombocytes and leukocytes have shown to normalize after treatment with praziquantel [4,6,19].

These haematological changes might not be unique to African people living in endemic areas and might be observable in general across geographical areas, age groups, and across *Schistosoma* species, and should possibly thus be observable among both migrants and travellers returning with schistosomiasis from endemic countries. This could be relevant, as the effects of *Schistosoma* species on the FBC might have the potential to be utilised as a surrogate diagnostic parameter [3] particularly in those where schistosomiasis might not be primarily suspected on the grounds of signs and symptoms or past medical history. We therefore investigated the effects of active (egg producing) *Schistosoma* infections on the FBC of returned travellers and migrants by performing a retrospective record review on patients that visited travel centres across Western Europe.

## 2 Methods

### 2.1 Patient selection

A retrospective patient record review was performed on *Schistosoma* infected migrants and returned travellers from miscellaneous geographical areas, as seen by seven European travel clinics located in Amsterdam, Barcelona, Berlin, Bordeaux, Madrid, Munich, and Negrar-Verona. Patient records were individually screened by the travel clinics applying a standardised data collection form. Cases were included if *Schistosoma* eggs of any species were detected in urine and/or faeces and if an FBC was performed. The following data items were collected: age; sex; migrant/traveller status; Hb level; erythrocytes; haematocrit; MCV; thrombocytes; total leukocytes; eosinophils; basophils; neutrophils; lymphocytes; monocytes; schistosomiasis serology; co-infection with other helminths; and concomitant diagnoses. Cases were excluded if the blood count had been assessed more than one month before or more than two weeks after detection of *Schistosoma* eggs, or if any concomitant diagnosis was present that could possibly or likely affect the FBC. Cases were not excluded if the concomitant diagnoses were a consequence of schistosomiasis (e.g. hepatosplenic schistosomiasis).

### 2.2 Outcomes and data analysis

Continuous data were presented in means and standard deviation (SD) and, if appropriate, in median and ranges; qualitative data were presented as proportions. The normality of the distribution of the data was determined by visual inspection. Not normally distributed data was log-transformed. If after log-transformation of not normally distributed data, a non-normal distribution remained, non-parametric tests were used for analysis. As a *Schistosoma*-negative control group was lacking, we decided to compare our results with FBC reference intervals used in the Central Diagnostic Laboratory of the Amsterdam UMC – location University of Amsterdam (CDL-AUMC).

A one-sample *t*-test was used to calculate the difference in means of FBC parameters of *Schistosoma* infected returned travellers and migrants, and the means of FBC reference values from the CDL-AUMC; a one-sample Wilcoxon signed rank test was used to compare medians for not-normally distributed data. In the case of ties, the average rank was used. The independent samples *t*-test was used to compare the means of two normally distributed groups, whilst the independent samples Mann-Whitney *U* Test was used for not-normally distributed data. To test for differences between more than two groups, the one-way ANOVA test was used for normally distributed continuous outcome data; the Kruskal-Wallis test was used for not-normally distributed continuous outcome data. The Pearson Chi-square test was used for binary outcome data. The level of statistical significance was set at less than 0.05, and we retained a 95% confidence interval (CI). Sub-analyses were performed for different age groups in children (as haematological reference values in children vary per age category), migrants and returned travellers, and types of schistosomiasis (urogenital, intestinal or both). Data analysis was carried out with IBM SPSS Statistics for Windows, version 28 (IBM Corp., Armonk, N.Y., USA). The data was presented in tables and in scatter plots using GraphPad Prism version 9.0.0 for Windows, GraphPad Software, San Diego, California USA, www.graphpad.com.

### 2.3 Ethics

No official approval of the study protocol led by the Dutch investigators was required, as the Medical Ethics Review Committee of the Academic Medical Centre confirmed that the Medical Research Involving Human Subjects Act (WMO) did not apply to our study, and an exemption letter was issued. For the German centres, the study was approved by the Ethics Committee of the Faculty of Medicine at LMU Munich (22–0129).

## 3 Results

### 3.1 Patient characteristics

A total of 655 patient records were obtained from *Schistosoma* infected patients, of which 273 were excluded, because the predefined time lapse between blood count and egg detection was exceeded, or because a concomitant diagnosis possibly or likely altering the FBC was established. The haematological outcomes of all returned travellers and migrants before exclusion of these concomitant diagnoses can be found in Table 1. An overview of all the concomitant diagnoses that were mentioned and their expected effect on the FBC is shown in Supplementary Table 5. A total of 382 *Schistosoma* infected returned travellers and migrants were eventually included in the analysis. Age ranged from 2 to 73 years, with a median of 21.0 years, and the majority of cases (85.1%) were men (Table 2). Sixty-six (17.3%) of the included S*chistosoma* infected cases were returned travellers, whereas the vast majority were all migrants (316; 86.7%). Most of the included cases were diagnosed with *S. haematobium* (164; 42.9%) or *S. mansoni* (194; 50.9%); 21 cases were infected with both, only one case with *S. intercalatum* and two cases with both *S. haematobium* and *S. intercalatum.*

**Table 1:** Haematological parameters of all *Schistosoma* infected returned travellers and migrants before exclusion of cases with concomitant diagnoses possibly or likely altering the FBC (n = 610) in comparison to CDL-AMC reference values.

*FBC parameter*	*Schistosoma* positive	Reference value		Difference in means	
	Mean (SD)	Mean	Normal range	Mean difference (95% CI)	Two-sided p-value∗
Haemoglobin (g/dL)	M: 14.7 (1.57)	M: 15.3	13.7–16.9	M: 0.6 (−0.71, −0.44)	**<0.001**
	F: 13.1 (1.36)	F: 14.1	12.1–16.1	F: 1.0 (−1.31, −0.64)	**<0.001**
Erythrocytes (10^6^/μL)	M: 5.22 (0.531)	M: 5.0	M: 4.5–5.5	M: +0.22 (0.179, 0.271)	**<0.001**
	F: 4.66 (0.459)	F: 4.5	F: 4.0–5.0	F: +0.16 (0.044, 0.279)	**0.008**
Haematocrit (%)	M: 44.3 (4.10)	M: 45	M: 40 - 50	M: 0.7 (−1.06, −0.35)	**<0.001**
	F: 39.3 (2.99)	F: 40	F: 35 - 45	F: 0.7 (−1.44, 0.10)	0.085
MCV fL	85.2 (6.95)	90	80–100	−4.8 (−5.39, −4.27)	**<0.001**
Thrombocytes (10^3^/μL)	227 (72.4)	275	150–400	−48 (−53.8, −42.2)	**<0.001**
Leukocytes (10^3^/μL)	5.83 (5.684, 5.980)^a^	7.25	4.0–10.5	−1.42 (−1.566, −1.270)^b^	**<0.001**
Eosinophils (10^3^/μL)	0.50 (0.465, 0.543)^a^	0.25	0–0.5	+0.25 (0.215, 0.293)^b^	**<0.001**
Basophils (10^3^/μL)	0.06 (0.053, 0.062)^a^	0.10	0–0.2	−0.04 (−0.047, −0.038)^b^	**<0.001**
Neutrophils (10^3^/μL)	2.28 (2.192, 2.362)^a^	4.50	1.8–7.2	−2.22 (−2.308, −2.138)^b^	**<0.001**
Lymphocytes (10^3^/μL)	2.31 (0.876)	2.75	1.5–4.0	−0.44 (−0.507, −0.365)	**<0.001**
Monocytes (10^3^/μL)	0.45 (0.198)	0.55	0.1–1.0	−0.10 (−0.113, −0.081)	**<0.001**

P-values in **bold** are statistically significant. ∗p-values are from a one-sample *t*-test. ^a^ Geometric mean (95% confidence interval); ^b^ Difference from geometric mean (95% confidence interval).

**Table 2:** Characteristics of *Schistosoma* infected returned travellers and migrants included for analysis.

	Total	Returned travellers	Migrants	p-value
Sample size	382	66 (17.3%)	316 (82.7%)	
Sex				
Male	325 (85.1%)	40 (60.6%)	285 (90.2%)	**<0.001∗**
Female	57 (14.9%)	26 (39.4%)	31 (9.8%)	
Age (median, range)	21.0 (2–73)	29 (2–73)	20.5 (3–54)	**<0.001ˆ**
Urogenital schistosomiasis	164 (42.9%)	38 (57.6%)	126 (39.9%)	**0.008∗**
Intestinal schistosomiasis^a^	195 (51.0%)	27 (40.9%)	168 (53.2%)	0.070**∗**
Mixed infection^b^	23 (6.0%)	1 (1.5%)	22 (7.0%)	0.091**∗**
Concomitant diagnoses:				
Complicated (hepato-) splenic Schistosomiasis	4 (1.0%)	0 (0%)	4 (1.3%)	0.358**∗**
Katayama syndrome	4 (1.0%)	4 (6.1%)	0 (0%)	**<0.001∗**
Ureteral stenosis	3 (0.8%)	2 (3.0%)	1 (0.3%)	0.460**∗**

P-values in **bold** are statistically significant. ∗p-values obtained by Pearson Chi-square test; ˆp-value obtained by Mann-Whitney *U* test. ^a^ 194 cases infected with S. mansoni and one with S. intercalatum; ^b^ 21 cases infected with S. mansoni and S. haematobium and two cases with S. haematobium and S. intercalatum.

### 3.2 Haematological outcomes in adults

The FBC of *Schistosoma* infected returned travellers and migrants of 16 years and older and their difference in means (β) from CDL-AMC reference values are shown in Table 3a. The mean Hb of infected patients was lower in both males and females than the mean of the reference values (15.0 *vs* 15.3 g/dL; *p-*value <0.001 and 13.1 vs 14.1 g/dL, *p-*value <0.001, respectively). Erythrocyte counts were slightly higher, reaching statistical significance in males, but not in females (5.27 vs 5.0 × 10^6^/μL, *p-*value <0.001 and 4.63 vs 4.5 × 10^6^/μL, *p-*value = 0.080, respectively). No significant differences were found in haematocrit (β = −0.1%, *p*-value = 0.694 [males], β = −0.6%, *p*-value = 0.232 [females]). The MCV was decreased (β = −4.2 fL, *p-*value <0.001). Thrombocytes were significantly lower than the mean of the reference value (225 vs 275 × 10^3^/μL, *p-*value <0.001). Furthermore, lower total leukocyte counts were recorded (5.74 vs 7.25 × 10^3^/μL, *p-*valu*e* < 0.001). As expected, the mean eosinophil count was significantly higher than the mean of the reference value (0.47 × 10^3^/μL vs 0.25 × 10^3^/μL, *p*-value <0.001). The other differential leukocyte counts, including basophils, neutrophils, lymphocytes and monocytes, were all significantly lower than the mean of the reference value (β = −0.04, −2.19, −0.50, and −0.11 × 10^3^/μL, respectively, *p-*value for all <0.001).

**Table 3a:** Haematological parameters of *Schistosoma* infected returned travellers and migrants 16 years and older (n = 355) in comparison to CDL-AMC reference values.

*FBC parameter*	*Schistosoma* positive	Reference value		Difference in means	
	Mean (SD)	Mean	Normal range	*Mean difference* β *(95% CI)*	Two-sided p-value∗
Haemoglobin (g/dL)	M: 15.0 (1.34)	M: 15.3	13.7–16.9	M: 0.3 (−0.50, −0.18)	**<0.001**
	F: 13.1 (1.14)	F: 14.1	12.1–16.1	F: 0.10 (−1.28, −0.62)	**<0.001**
Erythrocytes (10^6^/μL)	M: 5.27 (0.539)	M: 5.0	M: 4.5–5.5	M: +0.27 (0.202, 0.329)	**<0.001**
	F: 4.63 (0.469)	F: 4.5	F: 4.0–5.0	F: +0.13 (−0.016, 0.276)	0.080
Haematocrit (%)	M: 44.9 (3.55)	M: 45	M: 40 - 50	M: 0.1 (−0.50, 0.33)	0.694
	F: 39.4 (3.15)	F: 40	F: 35 - 45	F: 0.6 (−1.57, 0.39)	0.232
MCV fL	85.8 (6.49)	90	80–100	−4.2 (−4.93, −3.55)	**<0.001**
Thrombocytes (10^3^/μL)	225 (67.8)	275	150–400	−50 (−57.6, −43.3)	**<0.001**
Leukocytes (10^3^/μL)	5.74 (5.556, 5.935)^a^	7.25	4.0–10.5	−1.51 (−1.694, −1.315)^b^	**<0.001**
Eosinophils (10^3^/μL)	0.47 (0.428, 0.515)^a^	0.25	0–0.5	+0.22 (0.178, 0.265)^b^	**<0.001**
Basophils (10^3^/μL)	0.06 (0.055, 0.066)^a^	0.10	0–0.2	−0.04 (−0.045, −0.034)^b^	**<0.001**
Neutrophils (10^3^/μL)	2.30 (2.194, 2.415)^a^	4.50	1.8–7.2	−2.19 (−2.306, −2.085)^b^	**<0.001**
Lymphocytes (10^3^/μL)	2.25 (0.786)	2.75	1.5–4.0	−0.50 (−0.583, −0.416)	**<0.001**
Monocytes (10^3^/μL)	0.44 (0.199)	0.55	0.1–1.0	−0.11 (−0.131, −0.089)	**<0.001**

P-values in **bold** are statistically significant. ∗p-values obtained by a one-sample *t*-test. ^a^ Geometric mean (95% confidence interval); ^b^ Difference from geometric mean (95% confidence interval).

### 3.3 Haematological outcomes in children

The sample of children of seven to fifteen years of age was small, amounting to 24 subjects; 14 with urogenital schistosomiasis, nine with intestinal schistosomiasis and one with both. Haematocrit was significantly decreased (β = −2.5%, *p*-value = 0.018), whilst no significant differences were found in Hb, erythrocytes, MCV and thrombocytes. Similar to adults, decreases in leukocytes (β = −2.72 × 10^3^/μL, *p*-value <0.001) and differential leukocyte counts were found (basophils β = −0.05 × 10^3^/μL, *p-*value = 0.007; neutrophils β = −2.27 × 10^3^/μL, *p-*value < 0.001; lymphocytes β = −0.91 × 10^3^/μL, *p-*value <0.001; monocytes β = −0.08 × 10^3^/μL, *p-*value = 0.087); with an exception of eosinophils, which were increased (β = +0.30 × 10^3^/μL, *p-*value <0.001) (Table 3b). Only three children under the age of seven years were included, whose haematological outcomes are shown in Table 3c. No statistical tests were performed as the sample size was too small.

**Table 3b:** Haematological parameters of *Schistosoma* infected children 7–15 years of age (n = 24) in comparison to age-specific CDL-AMC reference values.

*FBC parameter*	*Schistosoma* positive	Reference value		Difference in means	
	Mean (SD)	Mean	Normal range	*Mean difference* β *(95% CI)*	Two-sided p-value∗
Haemoglobin (g/dL)	13.3 (1.710)	13.3	10.5–16.1	0.0 (−0.74, 0.70)	0.953
Erythrocytes (10^6^/μL)	4.80 (0.417)	4.7	3.8–5.6	+0.10 (−0.080, 0.272)	0.272
Haematocrit (%)	40.0 (4.55)	42.5	35–50	−2.5 (−4.50, −0.47)	**0.018**
MCV fL	82.8 (6.92)	85	75–95	−2.2 (−5.26, +0.88)	0.152
Thrombocytes (10^3^/μL)	271 (90.72)	300	150–450	−29 (−69.7, 10.7)	0.142
Leukocytes (10^3^/μL)	6.28 (1.649)	9.0	4.0–14.0	−2.72 (−3.416, −2.023)	**< 0.001**
Eosinophils (10^3^/μL)	0.55 (0.372, 0.803)^a^	0.25	0–0.5	+0.30 (0.122, 0.553)^b^	**<0.001**
Basophils (10^3^/μL)	0.05 (0.00, 0.40)^c^	0.10	0–0.2	−0.05^d^	**0.007ˆ**
Neutrophils (10^3^/μL)	2.48 (1.966, 3.125)^a^	4.75	1.5–8.0	−2.27 (−2.784, −1.625)^b^	**<0.001**
Lymphocytes (10^3^/μL)	2.09 (1.812, 2.403)^a^	3.0	1.0–5.0	−0.91 (−1.188, −0.597)^b^	**<0.001**
Monocytes (10^3^/μL)	0.47 (0.218)	0.55	0.1–1.0	−0.08 (−0.180, 0.013)	0.087

P-values in **bold** are statistically significant. ∗p-values obtained by a one-sample *t*-test; ˆ*p*-value from One-Sample Wilcoxon Signed Rank Test. ^a^ Geometric mean (95% confidence interval); ^b^ Difference from geometric mean (95% confidence interval); ^c^ Median (range); ^d^ Difference in medians.

**Table 3c:** Haematological parameters of *Schistosoma* infected children <7 years of age (n = 3).

FBC parameter	*Schistosoma* positive	Reference values	
	Median (Range)	6 months - 3 years Median (normal range)	3–7 years Median (normal range)
Haemoglobin (g/dL)	12.8 (10.8–13.4)	12.1 (9.7–14.5)	12.1 (9.7–14.5)
Erythrocytes (10^6^/μL)	4.79 (4.07–4.95)	4.4 (3.5–5.3)	4.4 (3.5–5.3)
Haematocrit (%)	39.0 (32.8–40.0)	36 (30–42)	36 (30–42)
MCV fL	81.0 (80.6–81.4)	77.5 (70–85)	80 (70–90)
Thrombocytes (10^3^/μL)	354 (154–372)	375 (150–600)	375 (150–600)
Leukocytes (10^3^/μL)	8.78 (6.94–11.41)	10 (4.0–16.0)	9.5 (4.0–15.0)
Eosinophils (10^3^/μL)	0.82 (0.59–1.14)	0.4 (0.0–0.8)	0.4 (0.0–0.8)
Basophils (10^3^/μL)	0.04 (0.00–0.05)	0.1 (0.0–0.2)	0.1 (0.0–0.2)
Neutrophils (10^3^/μL)	2.72 (1.23–4.91)	5.0 (1.0–9.0)	5.25 (1.5–9.0)
Lymphocytes (10^3^/μL)	5.36 (3.11–6.26)	4.75 (1.5–8.0)	3.5 (1.0–6.0)
Monocytes (10^3^/μL)	0.41 (0.00–0.48)	0.55 (0.1–1.0)	0.55 (0.1–1.0)

### 3.4 Haematological outcomes in returned travellers and migrants

**Fig. 1:**
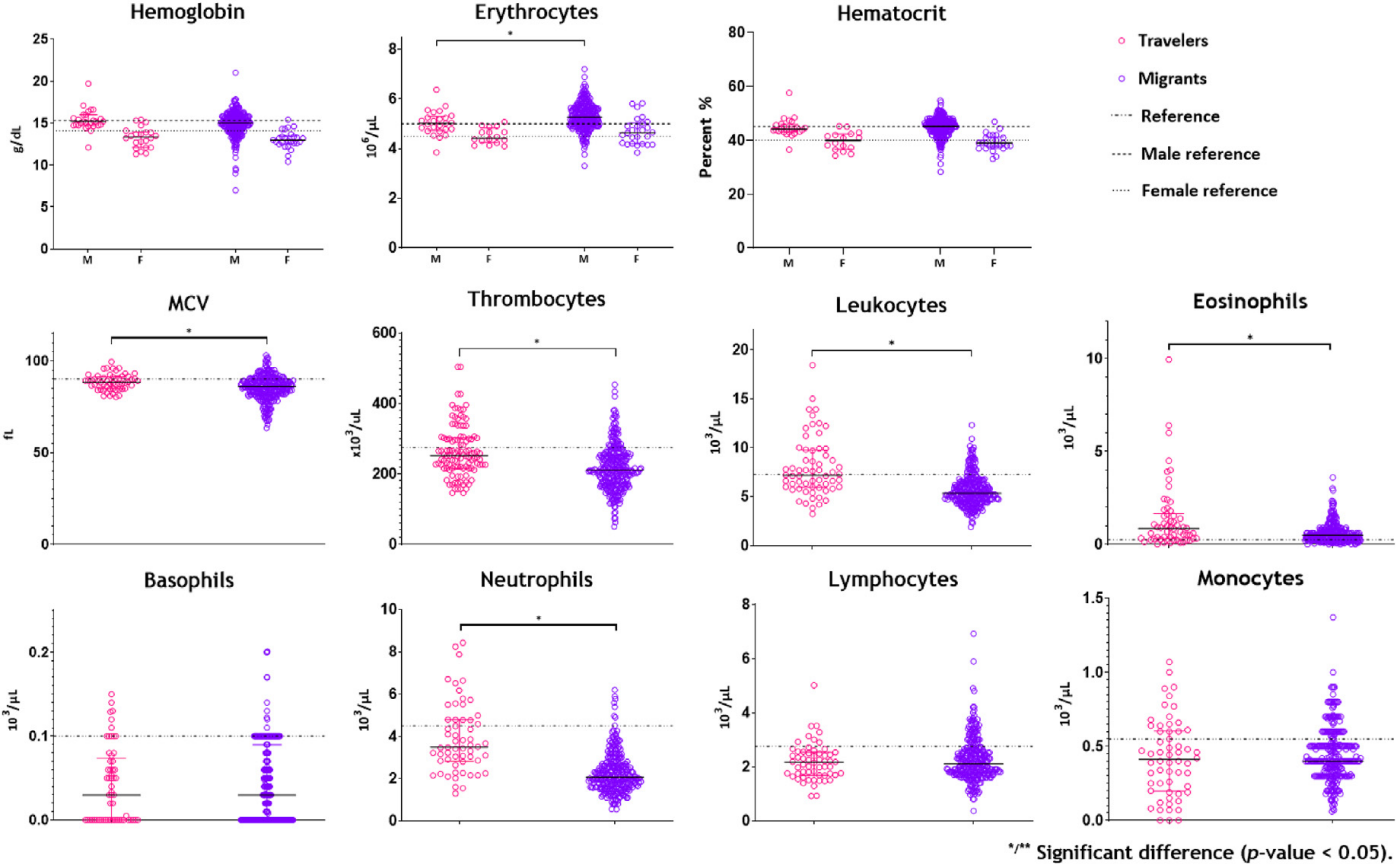
Haematological parameters of *Schistosoma* infected returned travellers versus migrants 16+ year-old in relation to CDL-AMC reference values. An independent samples *t*-test was used to test for differences in means between groups of normally distributed haematological parameters. An independent samples Mann-Whitney *U* Test was used for not-normally distributed haematological parameters.

Erythrocytes in males were significantly lower in returned travellers compared to migrants (β = −0.26 10^6^/μL, p-value = 0.023), whilst MCV was significantly higher (β = +3.1 fL, *p*-value <0.001). No significant differences were found in the other red blood cell parameters. Thrombocytes were significantly higher in returned travellers compared to migrants (β = +48 10^3^/μL, *p*-value <0.001). Leukocytes were higher as well (β = +2.35 10^3^/μL, *p*-value <0.001), accompanied by higher eosinophil and neutrophil counts (β = +0.26 10^3^/μL, *p*-value = 0.009 and β = +1.66 10^3^/μL *p*-value <0.001, respectively) (Fig. 1 and Supplementary Table 6a). When comparing returned travellers FBCs to the CDL-AMC reference values, significantly lower Hb in females (β = −0.82 g/dL, *p* = 0.005), and lower MCV were found (β = −1.6 fL, *p*-value = 0.009). Furthermore, significantly lower differential leukocyte counts were found, with an exception for eosinophils, that were significantly higher (β = +0.45 10^3^/μL, *p*-value <0.001) (Supplementary Table 6b). We refrained from including an analysis comparing migrants to the CDL-AMC, as these reference values might not appropriately be applicable to a heterogenous migrant population from across the globe.

### *3.5* *Haematological outcomes* per Schistosoma *species*

**Fig. 2:**
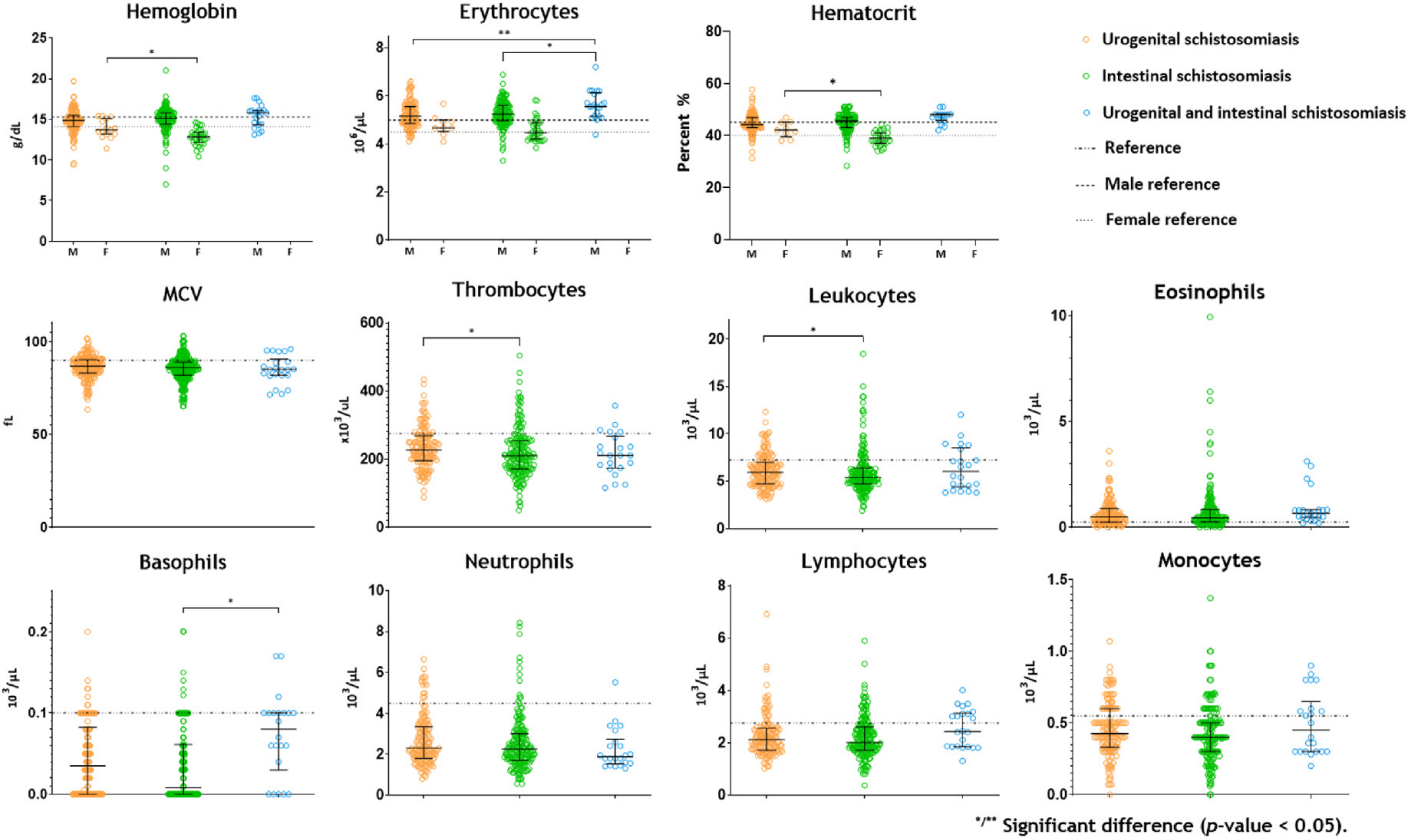
Haematological parameters of returned travellers and migrants 16 years-old infected with urogenital schistosomiasis, intestinal schistosomiasis or mixed in relation to CDL-AMC reference values. The one-way ANOVA was used to test for overall difference between groups; for not-normally distributed haematological parameters the Kruskal-Wallis test was used. In case of significant differences, an independent samples *t*-test were performed to explore which groups differed and in case of non-normally distributed data, an independent-samples Mann-Whitney *U* Test.

Certain differences in FBC were observed when comparing *Schistosoma* species (Fig. 2). Hb and haematocrit in females were significantly lower in intestinal schistosomiasis as compared to urogenital schistosomiasis (β = −1.0 g/dL, *p*-value = 0.003 and β = −3.7%, *p*-value = 0.007, respectively). Erythrocytes in males were higher when infected with two *Schistosoma* species than with one *Schistosoma* species (β = +0.41 10^6^/μL, *p*-value = 0.007 [urogenital] and β = +0.37 10^6^/μL, *p*-value = 0.013 [intestinal]). Additionally, thrombocytes and leukocytes were both significantly lower in intestinal schistosomiasis than in urogenital schistosomiasis (β = −15 10^3^/μL, *p*-value = 0.042 and β = −0.53 10^3^/μL, *p*-value = 0.023, respectively), and basophils were significantly lower in intestinal schistosomiasis in comparison to cases with both intestinal and urogenital schistosomiasis (β = −0.7 10^3^/μL, *p*-value = 0.002). The FBCs per *Schistosoma* species in comparison to the CDL-AMC reference values are shown in Supplementary Tables 7a–c, for urogenital schistosomiasis, intestinal schistosomiasis, and a combination of urogenital and intestinal schistosomiasis, respectively.

## 4 Discussion

This study aimed to determine if FBC differences in schistosomiasis might be so characteristic that a particular pattern could alert clinicians to the possibility of an active schistosomiasis, irrespective of a suggestive clinical picture and patient history.

In line with previous studies [[3], [4], [5], [6], [7], [8], [9],^11^,^12^], we found decreases in Hb and MCV that were significant in the analysis of individuals older than 16 years as well as in the sub-analyses of urogenital and intestinal schistosomiasis, whilst differences in haematocrit were not significant. Notably, we found significantly higher erythrocytes in males when individuals older than 16 years were analysed together. However, this increase in erythrocytes was not found in returned travellers, but only in migrants. Possibly, the reference value for erythrocytes from the CDL-AMC was too low to compare with erythrocytes of migrants, as erythrocyte reference values obtained from a study on populations from sub-Saharan African countries (where *Schistosoma*-positive are often migrants), were slightly higher and were actually comparable with the erythrocytes found in this cohort's male migrants [20].

Contrary to most earlier studies [3,5,7,9], we did not find higher, but rather significantly lower thrombocytes among individuals older than 16 years. In the sub-analyses, thrombocytes were significantly lower in migrants than in returned travellers. This could be due to the fact that in migrants the moment of exposure often dates back longer, thus they are more like to have schistosomiasis that may have progressed to the hepatosplenic stage, which is associated with low platelet counts, as a result of hypersplenism and portal hypertension (this was also observed in our dataset) [10,[16], [17], [18]]. However, although the decline in platelets was more pronounced in intestinal schistosomiasis, lower counts were also observed among individuals with urogenital schistosomiasis, in which hepatosplenic schistosomiasis did not play a role. One explanation might be that dual infections are overlooked as *S. mansoni* infection could be more difficult to diagnose, due to patients often not complying with turning in stool samples, and the sensitivity of stool microscopy being unsatisfactory.

Furthermore, we found leukocytes to be significantly lower among different age groups and in both intestinal and urogenital schistosomiasis, with decreases in all differential leukocyte counts; except for eosinophils, in which, as expected, significant increases were found. This is in line with earlier studies on Nigerian and Ethiopian adults, that observed significantly lower white blood cell counts in *S. mansoni* egg-positive than in *S. mansoni* egg-negative individuals [8,11,12]. One explanation for this phenomenon in *S. mansoni* infected people might be hypersplenism, which can lead to leukocyte destruction or sequestration in the spleen [21]. Two studies on urogenital schistosomiasis in Gabonese and Sudanese schoolchildren, on the other hand, found significant increases in the mean counts of all differential leukocyte types [3,4]. This is in contrast to our results in urogenital schistosomiasis, which calls for further exploration.

Strengths of our study include the large sample size and its representativeness for schistosomiasis patients across geographical areas, age groups, and *Schistosoma* species. A notable limitation of our study is the lack of an uninfected or egg-negative control group. Thereby, we had to compare our results with standard FBC reference values, for which we choose the one from the Amsterdam UMC – location AMC, that might not be representative for migrants from all over the world [20,22]. Although we excluded all cases with concomitant diagnoses, it remains uncertain how specific the observed FBC differences are, which also renders pathophysiological reasoning explaining that phenomenon difficult. Furthermore, we were only able to include a small number of children into our study and were thus unable to analyse haematological outcomes in children under the age of seven. To that end, unfortunately, we could not establish if the haematological changes found in African schoolchildren [[3], [4], [5], [6],^19^,[23], [24], [25], [26], [27]] are also present in this age group among returned travellers and migrants. Another limitation of our study is the relatively small number of females; whilst the vast majority of the study population were males. This gender imbalance might be explained by the fact that most study participants were migrants, and most migrants being men. Moreover, due to the retrospective study design, certain factors that might influence the effect of schistosomiasis on the FBC could not be taken into account. These include the intensity of the infection, as eggs were usually not quantified, and the duration of infection, which is often uncertain, as many patients are asymptomatic, and the moment of exposure is not always known. The vast majority of our study population were migrants, in which the time point of exposure could have been dating back years. Thus, the disease could have progressed to chronic organ damage stage, which itself – apart from active egg production might trigger some of the observed FBC alterations. Additionally, other unmeasured factors, such as nutrition status and undiagnosed current or past conditions, including co-infections that are reported to frequently occur, such as *Salmonella* [28], could also have impacted the FBC.

Although most studies agree that most red blood cell parameters tend to be decreased in schistosomiasis, observations on alterations in thrombocytes and leukocytes appear to vary between studies, showing either an increase or a decline. These differences in results between studies might best be understood if they are examined together in a systematic review and quantified in a meta-analysis. Moreover, further research should be undertaken to investigate the differences in FBC between active and progressed schistosomiasis infections. Ideally, age and sex-matched schistosomiasis-negative control groups would be included. Additionally, further investigations should be conducted particularly in young children and females to establish if haematological changes are also present among those groups not living in endemic countries.

## 5 Conclusion

Active egg-producing *Schistosoma* infections are associated with haematological alterations, which slightly differ between returned travellers and migrants, and between *Schistosoma* species. FBC alterations in both migrants and returned travellers include decreases in Hb, MCV, and all differential leukocyte counts; except for eosinophils, in which, as to be expected, increases were found. In migrants, lower thrombocyte and leukocyte counts were found, which were not present among returned travellers, which could have to do with a more advanced disease stage. However, the observed haematological differences were discrete and inhomogeneous, rendering them inapt to base a scoring system on. Hence, the FBC seems unsuitable as a surrogate diagnostic parameter to detect schistosomiasis in returned travellers and migrants.

## Funding

This research did not receive any specific grant from funding agencies in the public, commercial, or not-for-profit sectors.

## CRediT authorship contribution statement

**Jenny L. Schnyder:** Investigation, Methodology, Formal analysis, Writing – original draft. **Federico Gobbi:** Investigation, Writing – review & editing. **Mirjam Schunk:** Investigation, Writing – review & editing. **Andreas Lindner:** Investigation, Writing – review & editing. **Fernando Salvador:** Investigation, Writing – review & editing. **Alexandre Duvignaud:** Investigation, Writing – review & editing. **Marta Arsuaga Vicente:** Investigation, Writing – review & editing. **Jean Claude Dejon Agobé:** Writing – review & editing. **Paolo Cattaneo:** Investigation, Writing – review & editing. **Giulia Bertoli:** Investigation, Writing – review & editing. **Camilla Rothe:** Investigation, Writing – review & editing. **Mia Wintel:** Investigation, Writing – review & editing. **Diana Pou:** Investigation, Writing – review & editing. **Denis Malvy:** Investigation, Writing – review & editing. **Ayola Akim Adegnika:** Writing – review & editing. **Hanna K. De Jong:** Writing – review & editing. **Martin P. Grobusch:** Conceptualization, Methodology, Supervision, Writing – review & editing.

## Declaration of competing interest

All authors report no conflicts of interests.
