# Supplementary material for: Can haematological changes constitute a surrogate diagnostic parameter to detect schistosomiasis in migrants and travellers? - A retrospective analysis
Source: New Microbes New Infect. 2023 Apr 27;53:101136. doi: 10.1016/j.nmni.2023.101136 (PMC10176249; doi:10.1016/j.nmni.2023.101136)
Supplement: Multimedia component 1 [file mmc1.docx]

# **Supplementary Material**

**Supplementary Table 1**. Study characteristics and outcomes of studies on haematological differences of *Schistosoma mansoni.*

| **Name, year** | **Country** | **Study population** | **Intensity of infection*** | **N of egg positives** | **N of egg negative controls** | **Outcomes** |
| --- | --- | --- | --- | --- | --- | --- |
| Sturrock, 1996 [1] | Kenya | Schoolchildren from three primary schools the Miu Valley and the Kibwezi Division | Geometric mean of 198, 446 and 32 eggs/g, per school | 474 | NA | Before vs after PZQ:  Overall: Hb 12.36, Hct 40.50%, MCHC 30.63% vs Hb 12.12, Hct 40.38%, MCHC 30.03% |
| Nagi, 1999 [2] | Yemen | Schoolchildren in the Sana’a, Sada’a and Hajja governorate, 84.5% male, 82% 10-20 years old | Average egg count: 95 eggs/g of stool; in 75% egg count was below 100 eggs/g | 60 | NA | Egg-pos:  Hb 15.6  TWBC 6.0  Neutrophils (%) 38  Lymphocytes 49  Eosinophils 10  Monocytes 3  Basophils 0 |
| Butler, 2012 [3] | Kenya | Children attending public schools within 6 km of Lake Victoria, 9-12 years old | Low: 70  Moderate: 15  Heavy: 21 | 106 | 100 | S Mansoni low, moderate, heavy infection vs controls:  Hb 11.9 (11.5–12.3) 11.3 (10.2–12.5) 10.9 (9.9–11.9) vs 11.6 (11.3–12) g/dL |
| Elmissbah Elmahdi, 2013 [4] | Sudan | 77 males, 23 females, no other characteristics described | Not reported | 100 | 50 | Egg-pos vs egg-neg (means):  Males:  Hb 13.052 vs 14.480 g/dL  PCV 40.816 vs 39.750%  MCV 78.242 vs 85.530 fl  RBCs count 5.2284 vs 4.9460 x10^6^/uL  MCH 25.023 vs 29.070 pg  MCHC 31.994 vs 33.400 g/dL  Leukocyte count 7.4065 vs 6.6100 10^3^/uL  Platelet count 245.2626 vs 265.4000 10^3^/uL  Females:  Hb 12.052 vs 12.109 g/dL  PCV 37.796 vs 38.773%  MCV 74.817 vs 83.191 fl  RBCs count 5.0335 vs 4.6727 x10^6^/uL  MCH 23.835 vs 26.009 pg  MCHC 31.778 vs 31.155 g/dL  Leukocyte count 6.7522 vs 6.5091 10^3^/uL  Platelet count 240.0870 vs 341.2727 10^3^/uL |
| Egoro et al, 2017 [5] | Nigeria | People living in Ijora-Badia Lagos State, age range of 35-50 years | >2 eggs in stool | 35 | 35 | Egg-pos vs egg-neg:  Hb 7.0 vs 13.2 g/dL  WBC 2.5 vs 8.0 x10^3^/uL |
| Stecher, 2017 [6] | Mali | People living in the Segou region, aged 2-40 years | Not reported | 233 | 37 | Egg-pos vs egg-neg (means):  Hb 11.29±0.88 vs 12.59±0.32  Eosinophils 9.28 (1–30) vs 6.26 (0–32)  Platelets 256.00 (27–477) vs 234.45 (74–666) |
| Sorgho, 2017 [7] | Burkino Faso | People living in the Kou Valley, in the Houet province, age range 6-80, mean age 26.8, sex ratio M/F: 0.6 | Mild: 199  Moderate: 39  Heavy: 6 | 244 | 657 | Egg-pos vs egg-neg (means):  Hb: 11.57 vs 11.86 g/dL  Hct: 35.08 vs 35.97 %  RBC: 4.42 vs 4.40 x 10^6^/uL  MCV 79.55 vs 82.20  MCHC 26.26 vs 27.12  MCH 33.02 vs 32.98 g/dL  PLT 237.80 vs 225.93 x10^3^/uL  MPV 8.69 vs 8.36  WBC 6.92 vs 7.26 x10^3^/uL  Lymphocytes 3.24 vs 3.17 x10^3^/uL  Monocytes 0.68 vs 0.67 x10^3^/uL  Neutrophils 2.59 vs 2.28 x10^3^/uL  Eosinophils 0.37 vs 0.66 x10^3^/uL  Basophils 0.04 vs 0.05 x10^3^/uL |
| Eyayu, 2020 [8] | Ethiopia | People presenting themselves to Sanja Primary Hospital general outpatient department that were clinically suspected for intestinal parasite infection, median age of 27, 52% male | 0-99 epg  100-399 epg  >400 epg | 110 | 110 | Egg-pos vs egg-neg:  Thrombocytopenia 24/100 vs 3/100  Platelet count (median): 199 vs 268 x10^3^/uL  Light infection (0-99 epg): 211 x10^3^/uL; Moderate infection (100-399 epg): 202.5 x10^3^/uL; Heavy infection (>400 epg): 160 x10^3^/uL |
| Dessie, 2020 [9] | Ethiopia | People attending the Sanja hospital and healthy people from the Sanja community, mean age of 31, 50% male | Light 76 (69.1%) Moderate 31 (28.2) Heavy 3 (2.7%) | 110 | 110 | Egg-pos vs egg-neg:  Hb 11.36 vs 12.58;  RBC 3.92 vs 4.04;  PCV 34.14 vs 37.51;  MCV 92.78 vs 87.91;  MCH 28.90 vs 30.35;  MCHC 30.10 vs 34.80  WBC 4.60 vs 7.81;  Lymphocytes 1.50 vs 2.48;  mixed 0.55 vs 1.55;  granulocyte 2.63 vs 4.03; |

* Light infection: 1–99 EPG; moderate infection: 100–399 EPG; heavy infection: ≥400 EPG (World Health Organization. Prevention and control of schistosomiasis and soil-transmitted helminthiasis. 2003/02/21 ed. Geneva: World Health Organization; 2002. i–vi, 1–57, back cover p.)

**Supplementary Table 2.** Study characteristics and outcomes of studies on haematological differences of *Schistosoma haematobium.*

| **Name, year** | **Country** | **Study population** | **Intensity of infection*** | **N of egg positives** | **N of egg negative controls** | **Outcomes** |
| --- | --- | --- | --- | --- | --- | --- |
| Greenham, 1978 [10] | Kenya | Boys in Masabubu, Alinjurgur, Wajir Bor 10-16 years | Not specified | 67 | 69 | Egg-positive vs egg-negatives:  Hb < 8:  32/67 vs 4/69 |
| Wilkins, 1985 [11] | The Gambia | People living in Fajara, aged 2 years and older [] | Not described [] | 603 | 631 | \| Hb in egg-positives:  2-4 years: 9·4 (±1·8)  5-7 years: 10·6 (±1·7)  8-14 years: 11.6 (±1·5)  Males 15-44 years: 13·3 (±2·2)  Females 15-44 years:11.5 (±1·8)  Males 45+ years: 12·9 (±1·9)  Females 45+ years: 11·7 (±2·1) \| Hb in egg-negatives:  9.4 (±2·0)  11.1 (±1·5)  12·0 (±1·5)  14·2 (±1·8)  11.4 (±1·9)  13·2 (±1·7)  12·1 (±J.6) \| \| --- \| --- \| |
| Nagi, 1999 [2] | Yemen | Schoolchildren in the Sana’a, Sada’a and Hajja governorate, 84.5% male, 82% aged between 10-20 years | Not reported | 24 | NA | Egg-pos:  Hb 13.8  TWBC 6.1  Neutrophils (%) 38  Lymphocytes 53  Eosinophils 10  Monocytes 3  Basophils 0 |
| Bhargava, 2003 [12] | Tanzania | Schoolchildren in the coastal area of Bagamoyo and Kibaha districts, 9 to 15 years old | >50 eggs per 10 ml of urine, mean: 192.0 eggs/ 10 ml urine | Haematobium only:  79  Haematobium + hookworm: 135 | 116 | SH only, SH +HW vs controls:  Baseline:  Hb 11.44 vs 11.21 vs 11.9  Three months after treatment with PZQ and/or albendazol:  Hb 11.47, 11.56 vs 11.64  Fifteen months after treatment with PZQ and/or albendazol:  Hb 11.90, 12.09 vs 121.7 |
| Mohammed, 2006 [13] | Sudan | Schoolchildren, in El-Kriab village, 7 to 13 years old, with a mean body weight of 20 kg, 64 male and 33 female | Not reported | 50 | 20 | \| Before PZQ:  Hb 10.7 ± 0.2  Ht 31 ± 0.5  Leukocytes 10184 ± 30  Lymphocytes 321 ± 61  Neutrophils 3886 ± 89  Eosinophils 1572 ± 90  Monocytes 363 ± 59 \| After PZQ: 11 ± 0.2  33 ± 0.5  5260 ± 95 2374 ± 74 3645 ± 53 435 ± 50 139 ± 7 \| Control group: 11.2 ± 0.2  33 ± 0.7  5100 ± 82  2096 ± 58  2454 ± 76  359 ± 82  103 ± 27 \| \| --- \| --- \| --- \| |
| Stecher, 2017 [6] | Mali | People living in the Segou region, aged 2-40 years | 0.3-3.5 eggs per 10ml or urine: 77  3.5-22.5 eggs per 10ml or urine: 77  22.5-90.0 eggs per 10ml or urine: 75  >90.0 eggs per 10ml or urine:72 | 301 | 59 | \| 0.3-3.5 eggs:  Hb 12.71 ±0.56  Eosinophils  5.41 (0–18)  Platelets 220.23 (90–512) \| 3.5-22.5:  13.10±0.62  6.71 (0–20)  229.92 (27–477) \| 22.5-90:  12.18±0.61  7.07 (0–30)  245.45 (91–666) \| >90.0:  11.27±0.54  8.40 (1–32)  280.17 (116–664) \| Egg-neg  12.71±0.63  4.53 (0–14)  214.44 (81–364) \| \| --- \| --- \| --- \| --- \| --- \| |
| Afrifa, 2017 [14] | Ghana | Schoolchildren in Yeji, aged 6-17 years | Light: 37 subjects  Heavy: 13 subjects | 50 | 50 | \| Egg-positives:  WBC 7.80 ± 0.34  RBC 4.26 ± 0.08  Hb 9.99 ± 0.13  Ht 32.04 ± 0.46  MCV 75.87 ± 1.13  MCH 23.72 ± 0.43  MCHC 31.25 ± 0.27  Platelets 270.78 ± 13.93  RDW-CV 11.87 ± 0.21  Lymphocytes 49.20 ± 1.32  Mixed 13.49 ± 1.50  Neutrophils 40.79 ± 1.64 \| Egg-negatives:  7.26 ± 0.34  4.36 ± 0.06  11.36 ± 0.14  35.20 ± 0.42  79.71 ± 1.61  26.21 ± 0.31  32.21 ± 0.19  242.56 ± 9.93  11.21 ± 0.14  50.05 ± 1.41  9.16 ± 1.01  37.31 ± 1.70 \| \| --- \| --- \| |
| Dejon-Agobé, 2021 [15] | Gabon | School children living in Lambaréné, mean age of 10.1 (SD = 2.7) years, with a 0.95 female:male ratio. | Not reported | 451 | 161 | \| Egg-positives:  11.5, [10.7–12.1]  35.4, [32.7–36.6]  4.54, [4.30–4.85]  77.0, [73.0–81.0]  25.1, [23.7–26.5]  32.5, [31.8–33.2]  254, [195–315]  7.5, [6.30–9.10]  3.57, [3.11–4.40]  2.49, [2.04–3.19]  0.52, [0.33–0.90]  0.07, [0.05–0.09]  0.43, [0.01–0.61] \| Egg-negatives:  Hb 11.7 [11.1–12.3]  Ht 35.9 [34.2–35.8]  Erythrocytes 4.56 [4.29–4.83]  MCV 79.0 [75.0–83.0]  MCH 25.9 [24.4–27.5]  MCHC 32.7 [32.0–33.2]  Thrombocytes 232 [170–287]  Leukocytes 6.50 [5.50–8.00]  Lymphocytes 3.24 [2.64–4.40]  Neutrophils 2.29 [1.79–3.09]  Eosinophils 0.30 [0.16–0.50]  Basophils 0.05 [0.04–0.07]]  Monocytes 0.39 [0.03–0.61] \| \| --- \| --- \| |
| Sumbele, 2021 [16] | Cameroon | School children in Ikata, Bafia and Mile 14-Likoko in Muyuka, Cameroon, 4-14 years | Geometric mean egg count overall: 24 eggs/10 ml of urine | S Haematobium  99  SH + Pf malaria: 50 | 359 | \| Egg-pos:  Hb 10.5 (1.4)  Ht 30.3 (4.0)  WBC 9.3 (2.7)  RBC 4.2 (5.0)  Lymphocytes 3.8 (1.7)  MCV 73.2 (6.7)  MCH 25.3 (2.3)  MCHC 34.7 (1.8)  Platelet 279.0 (975.0)  RDW-CV% 12.7 (1.4) \| EP + malaria:  10.5 (1.3)  30.2 (3.7)  9.3 (3.0)  4.1 (4.7)  3.9 (2.1)  73.1 (6.1)  25.4 (2.4)  34.8 (2.4)  253.5 (118.7) 12.4 (1.2) \| Egg-neg:  10.8 (1.4)  30.9 (3.9)  9.9 (5.6)  4.2 (5.2)  4.3 (2.8)  73.4 (6.1)  25.5 (2.2)  35.0 (2.1)  280.9 (113.7) 12.5 (1.4) \| \| --- \| --- \| --- \| |

* Light infection: 1–49 eggs per 10 ml of urine; Heavy infection: ≥50 eggs per 10 ml. (World Health Organization. Prevention and control of schistosomiasis and soil-transmitted helminthiasis. 2003/02/21 ed. Geneva: World Health Organization; 2002. i–vi, 1–57, back cover p)

**Supplementary Table 3.** Differences in means of FBC parameters of *Schistosoma mansoni* infected African schoolchildren versus non-infected controls.

|  | Egoro et al, 2017 [5] | | Stecher et al, 2017 [6] | | | Sorgho et al 2017 [7] | | | Eyayu et al, 2020 [8] | | | | Dessie et al, 2020 [9] | |
| --- | --- | --- | --- | --- | --- | --- | --- | --- | --- | --- | --- | --- | --- | --- |
| FBC parameter | Difference  in means | p-value | | Difference  in means | p-value | | Difference  in means | p-value | | Difference  in means | p-value | Difference  in means | | p-value |
| Haemoglobin (g/dL) | **-6.2** | **<0.05** | | **-1.3** | **<0.01** | | **-0.29** | **0.02** | | NA |  | **-1.22** | | **<0.001** |
| Erythrocytes (10^6^/µL) | NA |  | | NA |  | | +0.02 | 0.684 | | NA |  | -0.12 | | 0.208 |
| Haematocrit (%) | NA |  | | NA |  | | **-0.89** | **0.017** | | NA |  | NA | |  |
| MCV (µm^3^) | NA |  | | NA |  | | **-2.65** | **0.023** | | NA |  | **+4.87** | | **<0.001** |
| MCH (pg) | NA |  | | NA |  | | +0.04 | 0.312 | | NA |  | **-1.45** | | **0.001** |
| MCHC (g/dL) | NA |  | | NA |  | | **-0.86** | **0.039** | | NA |  | **-4.70** | | **<0.001** |
| RDW (%) | NA |  | | NA |  | | NA |  | | NA |  | NA | |  |
| Platelet (10^3^/µL) | NA |  | | +21.55 | NA | | +11.87 | 0.993 | | **-69** | **<0.001** | NA | |  |
| Leukocytes (10^3^/µL) | **-5.5** | **<0.05** | | NA |  | | -0.34 | 0.575 | | NA |  | **-3.21** | | **<0.001** |
| Lymphocytes (10^3^/µL) | NA |  | | NA |  | | +0.07 | 0.434 | | NA |  | **-0.98** | | **<0.001** |
| Neutrophils (10^3^/µL) | NA |  | | NA |  | | +0.31 | 0.21 | | NA |  | **-1.4** | | **<0.001** |
| Eosinophils (10^3^/µL) | NA |  | | **+3.02** | **<0.05** | | -0.29 | 0.992 | | NA |  | NA | |  |
| Basophiles (10^3^/µL) | NA |  | | NA |  | | -0.01 | 0.849 | | NA |  | NA | |  |
| Monocytes (10^3^/µL) | NA |  | | NA |  | | +0.01 | 0.567 | | NA |  | NA | |  |

Significant differences in means are shown in **bold**.

**Supplementary Table 4.** Differences in means of FBC parameters of *Schistosoma haematobium* infected African schoolchildren versus non-infected controls.

|  | Dejon‑Agobé et al, 2021 [15]*  (N=161 vs 451) | | Mohammed et al, 2006 [13]  (N= 50 vs 20) | | | Afrifa et al, 2017 [14]  (N= 50 vs 50) | | | Bhargava et al, 2003 [12]  (N= 79 vs 116) | | | | Sumbele et al, 2021 [16]  (N= 99 vs 359) | |
| --- | --- | --- | --- | --- | --- | --- | --- | --- | --- | --- | --- | --- | --- | --- |
| FBC parameter | Difference  in means | p-value | | Difference  in means | p-value | | Difference  in means | p-value | | Difference  in means | p-value | Difference  in means | | p-value |
| Haemoglobin (g/dL) | −0.20 | 0.08 | | −0.50 | NS | | −**1.37** | **<0.0001** | | −0.46 | NA | −0.3 | | NS |
| Erythrocytes (10^6^/µL) | +0.006 | 0.89 | | NA |  | | −0.10 | 0.289 | | NA |  | 0 | | NS |
| Haematocrit (%) | −0.61 | 0.06 | | -2 | NS | | −**3.16** | **<0.0001** | | NA |  | −0.6 | | NS |
| MCV (µm^3^) | **−1.50** | **0.02** | | NA |  | | −**3.84** | **0.0053** | | NA |  | −0.2 | | NS |
| MCH (pg) | **−0.54** | **0.04** | | NA |  | | −**2.49** | **<0.0001** | | NA |  | −0.2 | | NS |
| MCHC (g/dL) | −0.02 | 0.83 | | NA |  | | −**0.96** | **0.005** | | NA |  | −0.3 | | NS |
| RDW (%) | NA |  | | NA |  | | **+0.66** | **0.012** | | NA |  | −0.1 | | NS |
| Platelet (10^3^/µL) | **+28.2** | **0.002** | | NA |  | | +28.22 | 0.102 | | NA |  | −1.9 | | NS |
| Leukocytes (10^3^/µL) | **+1.13** | **0.0003** | | **+5.084** | **<0.01** | | +0.54 | 0.264 | | NA |  | −0.6 | | NS |
| Lymphocytes (10^3^/µL) | **+1.11** | **0.001** | | −**1.775** | **<0.01** | | +0.20 | 0.661 | | NA |  | −0.5 | | NS |
| Neutrophils (10^3^/µL) | **+1.12** | **0.01** | | **+1.432** | **<0.01** | | +0.47 | 0.144 | | NA |  | NA | | NS |
| Eosinophils (10^3^/µL) | **+1.64** | **<0.001** | | **+1.213** | **<0.00** | | NA |  | |  |  | NA | |  |
| Basophiles (10^3^/µL) | **+1.27** | **0.0001** | | NA |  | | NA |  | |  |  | NA | |  |
| Monocytes (10^3^/µL) | NA |  | | **+0.260** | **<0.01** | | NA |  | |  |  | NA | |  |

Significant differences in means are shown in **bold**. * These results were adjusted on *Plasmodium falciparum* infection, any soil-transmitted helminthic infections, and use of praziquantel.

Abbreviations: PZQ: praziquantel; Hb: haemoglobin; Ht: haematocrit; RBC: red blood cell count; MCV: mean Corpuscular volume; MCH: Mean Corpuscular Haemoglobin; MCHC: Mean Corpuscular Haemoglobin Concentration; RDW: Red blood cell distribution width; WBC: white blood count.

**Supplementary Table 5.** Concomitant diagnoses mentioned in the database and their expected effect on the full blood count (FBC).

| **Likely or possibly affecting FBC, but diagnosis is (probably) consequence of schistosomiasis** | **Likely affecting FBC** | **Possibly affecting FBC** | **No significant effects on the FBC expected** |
| --- | --- | --- | --- |
| Hepatosplenic schistosomiasis:   - Including (hepato-) splenomegaly **(x9)** - Complicated by pre-portal hypertension and oesophageal varices **(x3)** - Complicated by liver cirrhosis **(x3)** | Tuberculosis **(x11):**   - Pulmonary tuberculosis **(x4)** - Miliary tuberculosis **(1x)** - Extra-pulmonary tuberculosis **(x1)** - Nodal tuberculosis **(x1)** - Complicated with severe fibrosis with multiple cavitations, cystic degeneration and hemoptysis **(x1)** | Co-infections with other helminths **(x115):**   - Strongyloidiasis **(x57)** - Hookworms **(x30)** - Filariasis **(x19)** - Trichiuriasis **(x7)** - Ascariasis **(x6)** - Hymenolepiasis **(x6)** - Taeniasis **(x4)** - Trichostrongyliasis **(x2)** | Latent tuberculosis infection **(x78)** |
| Ureteral stenosis caused by urogenital schistosomiasis **(x3)** | Malaria **(x17):**   - P. falciparum malaria **(x11)** - P. vivax malaria **(x1)** - P. malariae malaria **(x1)** | Hepatitis B **(x80):**   - Chronic hepatitis B infection **(21x)** - History of hepatitis B infection **(x15)** - Co-infection with hepatitis D **(x4)** - High viral load **(x5)** - Low viral load **(x15)** | Giardiasis **(x29)**  Amoebiasis **(x41)** |
| Lung schistosomiasis **(x1)** | Splenomegaly **(x4):**   - Hyper-reactive malarial splenomegaly **(x1)** - Tropical splenomegaly **(x1)** | (Acute) gastritis **(x14):**   - H. pylori infection **(x12)** | Latent syphilis **(x15)** |
| Katayama syndrome **(x4)** | Sickle cell anaemia **(x2)**, thalassaemia **(2x)** | HIV **(x10)** | Sickle cell trait **(x12)**  Haemoglobin C heterozygosis **(x2)** |
|  | Others:   - Iron deficiency **(x2)** - Pregnancy **(x1)** - Atypical bronchial carcinoid, stage IIIa (pT3N1M0) **(x1)** - Carcinoid tumour of the lung **(x1)** - Severe nephrotic syndrome of unclear origin with anasarca **(x1)** - Cholecystitis **(x1)** - Osteomyelitis **(x3)** - Syphilis **(x1)** | Others:   - Enteritis (Campylobacter **x2**; Salmonella **x1**) - Upper respiratory tract infection - Recurrent skin infections with PVL+ S. aureus (**x2**) - Elevated creatinine **(x4)** - Asthma **(x1)** - Pneumoconiosis **(x1)** - Hypothyroidism **(x1)** - Diabetes **(x1)** - Ischemia conus medullaris **(x1)** - Cachexia **(x1)** - Sarcoidosis **(x1)** | Others:   - Dissociative disorder **(x1)** - Pronounced degenerative changes in the thoracic and lumbar spine **(x1)** - Suspect unilateral renal agenesis **(x1)** - Pruritus of unknown origin **(x2)** - Toxoplasmic chorioretinitis **(x1)** - Tinea capitis **(x1)** - Elevated creatinine kinase **(x1)** - Past splenectomy **(x1)** - Scabies **(x2)** - Complicated inguinal hernia **(x1)** |

The number of cases per diagnosis reported in the database are shown in **bold.**

**Supplementary Table 6a.** Haematological parameters of *Schistosoma* infected returned travellers (n=294) versus migrants (n=61) 16+ year-old.

|  | **Returned travellers** | **Migrants** |  |  |
| --- | --- | --- | --- | --- |
|  | *Mean (SD)* | *Mean (SD)* | *Mean difference* β *(95% CI)* | *Two-sided p-value** |
| Haemoglobin (g/dL) | M: 15.2 (12.1-19.7)^a^  F: 13.3 (1.22) | M: 15.0 (9.0-17.8)^a^  F: 13.0 (1.07) | M: +0.2^b^  F: +0.2 (-0.42, 0.91) | 0.244^  0.2427 |
| Erythrocytes (10^6^/µL) | M: 5.03 (0.492)  F: 4.51 (0.314) | M: 5.29 (0.539)  F: 4.70 (0.536) | M: -0.26 (-0.478, -0.036)  F: -0.19 (-0.490, 0.108) | **0.023**  0.102 |
| Haematocrit (%) | M: 44.1 (36.5-57.6)^a^  F: 39.7 (3.43) | M: 45.0 (28.3-54.8)^a^  F: 39.2 (30.3) | M: -0.9^b^  F: +0.44 (-1.61, 2.48) | 0.252^  0.667 |
| MCV fL | 88.4 (4.31) | 85.3 (6.71) | +3.1 (1.73, 4.53) | **<0.001** |
| Thrombocytes (10^3^/µL) | 265 (74.1) | 217 (63.9) | +48 (28.6, 66.6) | **<0.001** |
| Leukocytes (10^3^/µL) | 7.99 (3.049) | 5.64 (1.595) | +2.35 (1.547, 3.149) | **<0.001** |
| Eosinophils (10^3^/µL) | 0.70 (0.500, 0.984)^c^ | 0.44 (0.396, 0.484)^d^ | +0.26 (0.062, 0.546)^d^ | **0.009** |
| Basophils (10^3^/µL) | 0.03 (0.00-0.15)^a^ | 0.03 (0.00-0.20)^a^ | 0.00^b^ | 0.879^ |
| Neutrophils (10^3^/µL) | 3.93 (1.671) | 2.274 (0.938) | +1.66 (1.206, 2.111) | **<0.001** |
| Lymphocytes (10^3^/µL) | 2.18 (0.692) | 2.26 (0.805) | -0.09 (-0.308, 0.137) | 0.452 |
| Monocytes (10^3^/µL) | 0.42 (0.262) | 0.44 (0.184) | -0.02 (-0.093, 0.051) | 0.554 |

P-values in **bold** are statistically significant. *p-values obtained by an independent samples t-test; ^p-value from independent samples Mann-Whitney U Test. ^a^ Median (range); ^b^ Difference in median; ^c^ Geometric mean (95% confidence interval); ^d^ Difference from geometric mean (95% confidence interval).

**Supplementary Table 6b.** Haematological parameters of *Schistosoma* infected returned travellers older than 16 years in comparison to CDL-AMC reference values (n = 61).

|  | **Returned travellers** | | | **Reference value** | | **Difference in means** | | |
| --- | --- | --- | --- | --- | --- | --- | --- | --- |
| FBC parameter | | *Mean (SD)* | *Mean* | | *Normal range* | | *Mean difference* β *(95% CI)* | *Two-sided p-value** |
| Haemoglobin (g/dL) | | M: 15.2 (12.1-19.7)^a^  F: 13.3 (1.22) | M: 15.3 F: 14.1 | | 13.7 - 16.9  12.1 - 16.1 | | M: -0.1^b^  F: -0.82 (-1.36, -0.28) | 0.770^  **0.005** |
| Erythrocytes (10^6^/µL) | | M: 5.03 (0.492)  F: 4.51 (0.314) | M: 5.0  F: 4.5 | | M: 4.5 - 5.5  F: 4.0 - 5.0 | | M: +0.03 (-0.172, 0.235)  F: +0.01 (-0.156, 0.179) | 0.751  0.882 |
| Haematocrit (%) | | M: 44.1 (36.5-57.6)^a^  F: 39.7 (3.43) | M: 45  F: 40 | | M: 40 - 50  F: 35 - 45 | | -0.9^b^  -0.3 (-2.15, 1.51) | 0.241^  0.715 |
| MCV fL | | 88.4 (4.31) | 90 | | 80-100 | | -1.6 (-2.78, -0.42) | **0.009** |
| Thrombocytes (10^3^/µL) | | 265 (74.1) | 275 | | 150 - 400 | | -10 (-30.5, 9.6) | 0.301 |
| Leukocytes (10^3^/µL) | | 7.99 (3.049) | 7.25 | | 4.0 - 10.5 | | +0.74 (-0.039, 1.523) | 0.062 |
| Eosinophils (10^3^/µL) | | 0.70 (0.500, 0.984)^c^ | 0.25 | | 0 - 0.5 | | +0.45 (0.250, 0.734)^d^ | **<0.001** |
| Basophils (10^3^/µL) | | 0.03 (0.00-0.15)^a^ | 0.10 | | 0 - 0.2 | | -0.07^b^ | **<0.001^** |
| Neutrophils (10^3^/µL) | | 3.93 (1.671) | 4.50 | | 1.8 - 7.2 | | -0.57 (-1.007, -0.128) | **0.012** |
| Lymphocytes (10^3^/µL) | | 2.18 (0.692) | 2.75 | | 1.5 - 4.0 | | -0.57 (-0.752, -0.389) | **<0.001** |
| Monocytes (10^3^/µL) | | 0.42 (0.262) | 0.55 | | 0.1 - 1.0 | | -0.13 (-0.197, -0.059) | **<0.001** |

P-values in **bold** are statistically significant. *p-values obtained by a one-sample t-test; *^p*-values from One-Sample Wilcoxon Signed Rank Test. ^a^ Median (range); ^b^ Difference in medians; ^c^ Geometric mean (95% confidence interval); ^d^ Difference from geometric mean (95% confidence interval).

**Supplementary Table 7a.** Haematological parameters of returned travellers and migrants 16+ year-old with urogenital schistosomiasis (S. haematobium) (n=150) in comparison to CDL-AMC reference values.

|  | ***Urogenital schistosomiasis*** | | | **Reference value** | | **Difference in means** | | |
| --- | --- | --- | --- | --- | --- | --- | --- | --- |
| FBC parameter | | *Mean (SD)* | *Mean* | | *Normal range* | | *Mean difference* β *(95% CI)* | *Two-sided*  *p-value** |
| Haemoglobin (g/dL) | | M: 14.8 (1.41)  F: 13.8 (1.16) | M: 15.3 F: 14.1 | | 13.7 - 16.9  12.1 - 16.1 | | M: -0.5 (-0.76, -0.25)  F: -0.3 (-0.89, 0.39) | **<0.001**  0.411 |
| Erythrocytes (10^6^/µL) | | M: 5.22(0.526)  F: 4.78 (0.445) | M: 5.0  F: 4.5 | | M: 4.5 - 5.5  F: 4.0 - 5.0 | | M: +0.22 (0.120, 0.310)  F: +0.28 (-0.063, 0.621) | **<0.001**  0.097 |
| Haematocrit (%) | | M: 44.8 (3.69)  F: 42.3 (3.07) | M: 45  F: 40 | | M: 40 - 50  F: 35 - 45 | | M: -0.2 (-0.92, 0.42)  F: +2.3 (-0.07, 4.65) | 0.464  0.055 |
| MCV fL | | 86.4 (6.37) | 90 | | 80-100 | | -3.6 (-4.68, -2.57) | **<0.001** |
| Thrombocytes (10^3^/µL) | | 234 (60.7) | 275 | | 150 - 400 | | -41 (-51.4, -31.4) | **<0.001** |
| Leukocytes (10^3^/µL) | | 6.12 (1.769) | 7.25 | | 4.0 - 10.5 | | -1.13 (-1.412, -0.841) | **<0.001** |
| Eosinophils (10^3^/µL) | | 0.46 (0.390, 0.538)^a^ | 0.25 | | 0 - 0.5 | | +0.21 (0.140, 0.288)^b^ | **<0.001** |
| Basophils (10^3^/µL) | | 0.06 (0.049, 0.065)^a^ | 0.10 | | 0 - 0.2 | | -0.04 (-0.051, -0.035)^b^ | **<0.001** |
| Neutrophils (10^3^/µL) | | 2.66 (1.203) | 4.50 | | 1.8 - 7.2 | | -1.84 (-2.042, -1.639) | **<0.001** |
| Lymphocytes (10^3^/µL) | | 2.17 (2.057, 2.281)^a^ | 2.75 | | 1.5 - 4.0 | | -0.58 (-0.693, -0.469)^b^ | **<0.001** |
| Monocytes (10^3^/µL) | | 0.45 (0.190) | 0.55 | | 0.1 - 1.0 | | -0.10 (-0.129, -0.066) | **<0.001** |

P-values in **bold** are statistically significant. *p-values obtained by a one-sample t-test. ^a^ Geometric mean (95% confidence interval); ^b^ Difference from geometric mean (95% confidence interval).

**Supplementary Table 7b.** Haematological parameters of infected returned travellers and migrants 16+ year-old with intestinal schistosomiasis (*S. mansoni* or *S. intercalatum*) (n=183) in comparison to CDL-AMC reference values.

|  | ***Intestinal schistosomiasis*** | | | **Reference value** | | **Difference in means** | | |
| --- | --- | --- | --- | --- | --- | --- | --- | --- |
| FBC parameter | | *Mean (SD)* | *Mean* | | *Normal range* | | *Mean difference* β *(95% CI)* | *Two-sided p-value* |
| Haemoglobin (g/dL) | | M: 15.0 (1.26)  F: 12.8 (0.989) | M: 15.3 F: 14.1 | | 13.7 - 16.9  12.1 - 16.1 | | M: -0.3 (-0.47, -0.05)  F: -1.3 (-1.62, -0.92) | **0.016**  **<0.001** |
| Erythrocytes (10^6^/µL) | | M: 5.26 (0.524)  F: 4.59 (0.474) | M: 5.0  F: 4.5 | | M: 4.5 - 5.5  F: 4.0 - 5.0 | | M: +0.26 (0.168, 0.344)  F: +0.09 (-0.079, 0.257) | **<0.001**  0.286 |
| Haematocrit (%) | | M: 44.9 (3.47)  F: 38.6 (2.72) | M: 45  F: 40 | | M: 40 - 50  F: 35 - 45 | | M: -0.1 (-0.73, 0.44)  F: -1.4 (-2.34, -0.41) | 0.620  **0.007** |
| MCV fL | | 85.4 (6.44) | 90 | | 80-100 | | -4.6 (-5.57, -3.67) | **<0.001** |
| Thrombocytes (10^3^/µL) | | 218 (73.2) | 275 | | 150 - 400 | | -57 (-67.5, -45.8) | **<0.001** |
| Leukocytes (10^3^/µL) | | 5.59 (5.325, 5.877) ^a^ | 7.25 | | 4.0 - 10.5 | | -1.66 (-1.925, -1.373)^b^ | **<0.001** |
| Eosinophils (10^3^/µL) | | 0.47 (0.403, 0.537)^a^ | 0.25 | | 0 - 0.5 | | +0.22 (0.153, 0.287)^b^ | **<0.001** |
| Basophils (10^3^/µL) | | 0.06 (0.051, 0.068)^a^ | 0.10 | | 0 - 0.2 | | -0.04 (-0.049, -0.032)^b^ | **<0.001** |
| Neutrophils (10^3^/µL) | | 2.24 (2.089, 2.399)^a^ | 4.50 | | 1.8 - 7.2 | | -2.26 (-2.411,-2.101)^b^ | **<0.001** |
| Lymphocytes (10^3^/µL) | | 2.19 (0.778) | 2.75 | | 1.5 - 4.0 | | -0.56 (-0.669, -0.442) | **<0.001** |
| Monocytes (10^3^/µL) | | 0.42 (0.202) | 0.55 | | 0.1 - 1.0 | | -0.13 (-0.156, -0.097) | **<0.001** |

P-values in **bold** are statistically significant. *p-values obtained by a one-sample t-test. ^a^ Geometric mean (95% confidence interval); ^b^ Difference from geometric mean (95% confidence interval).

**Supplementary Table 7c.** Haematological parameters of infected returned travellers and migrants 16+ year-old with both urogenital and intestinal schistosomiasis (*S. haematobium* and *S. mansoni* or *S. intercalatum*) (n=22) in comparison to CDL-AMC reference values.

|  | ***Urogenital and intestinal schistosomiasis*** | | | **Reference value** | | **Difference in means** | | |
| --- | --- | --- | --- | --- | --- | --- | --- | --- |
| FBC parameter | | *Mean (SD)* | *Mean* | | *Normal range* | | *Mean difference* β *(95% CI)* | *Two-sided p-value* |
| Haemoglobin (g/dL) | | M: 15.4 (1.33)  F: *NA* | M: 15.3 F: 14.1 | | 13.7 - 16.9  12.1 - 16.1 | | M: +0.1 (-0.47, 0.750)  F: *NA* | 0.629  *NA* |
| Erythrocytes (10^6^/µL) | | M: 5.62 (0.598)  F: *NA* | M: 5.0  F: 4.5 | | M: 4.5 - 5.5  F: 4.0 - 5.0 | | M: +0.62 (0.350, 0.895)  F: *NA* | **<0.001**  *NA* |
| Haematocrit (%) | | M: 46.3 (3.05)  F: *NA* | M: 45  F: 40 | | M: 40 - 50  F: 35 - 45 | | M: +1.3 (-0.123, 2.656)  F: *NA* | 0.072  *NA* |
| MCV fL | | 84.9 (7.59) | 90 | | 80-100 | | -5.1 (-8.48, -1.75) | **0.005** |
| Thrombocytes (10^3^/µL) | | 216 (61.9) | 275 | | 150 - 400 | | -59 (-86.6, -31.7) | **<0.001** |
| Leukocytes (10^3^/µL) | | 6.38 (2.33) | 7.25 | | 4.0 - 10.5 | | -0.87 (-1.907, 0.159) | 0.093 |
| Eosinophils (10^3^/µL) | | 0.69 (0.492, 0.970)^a^ | 0.25 | | 0 - 0.5 | | +0.44 (0.242, 0.720)^b^ | **<0.001** |
| Basophils (10^3^/µL) | | 0.07 (0.051) | 0.10 | | 0 - 0.2 | | -0.03 (-0.050, -0.005) | **0.021** |
| Neutrophils (10^3^/µL) | | 2.09 (1.769, 2.479)^a^ | 4.50 | | 1.8 - 7.2 | | -2.41 (-2.731, -2.021)^b^ | **<0.001** |
| Lymphocytes (10^3^/µL) | | 2.55 (0.743) | 2.75 | | 1.5 - 4.0 | | -0.20 (-0.529, 0.130) | 0.222 |
| Monocytes (10^3^/µL) | | 0.45 (0.371, 0.550) ^a^ | 0.55 | | 0.1 - 1.0 | | -0.10 (-0.179, 0.000) ^b^ | 0.051 |

P-values in **bold** are statistically significant. *p-values obtained by a one-sample t-test. ^a^ Geometric mean (95% confidence interval); ^b^ Difference from geometric mean (95% confidence interval).

**References**

[1] Sturrock R, Kariuki H, Thiongo F, Gachare J, Omondi B, Ouma J, et al. Schistosomiasis mansoni in Kenya: relationship between infection and anaemia in schoolchildren at the community level. Transactions of the Royal Society of Tropical Medicine and Hygiene. 1996;90(1):48-54.

[2] Nagi M, Kumar A, Mubarak J, Bamashmoos S. Epidemiological, clinical and haematological profile of schistosomiasis in Yemen. EMHJ-Eastern Mediterranean Health Journal. 1999;5(1):177-81.

[3] Butler SE, Muok EM, Montgomery SP, Odhiambo K, Mwinzi PM, Secor WE, et al. Mechanism of anemia in Schistosoma mansoni–infected school children in western Kenya. The American journal of tropical medicine and hygiene. 2012;87(5):862.

[4] ElmissbahElmahdi T, Mustafa AMHI. Measuring the impact of schistosomiasis infection on different blood parameters. Parasitology. 2013;7(3).

[5] Egoro ET, Ilegbedion GI, Loveday ZU, Shonibare MS. Blood biochemical and haematological alterations in Schistosoma mansoni infected patients in Ijora–Badia Nigeria. European Journal of Biomedical and Pharmaceutical Sciences. 2017;4(11):148-52.

[6] Stecher CW, Sacko M, Madsen H, Wilson S, Wejse C, Keita AD, et al. Anemia and growth retardation associated with Schistosoma haematobium infection in Mali: a possible subtle impact of a neglected tropical disease. Transactions of The Royal Society of Tropical Medicine and Hygiene. 2017;111(4):144-53.

[7] Sorgho H, Da O, Rouamba T, Savadogo B, Tinto H, Ouedraogo J-B. Schistosoma mansoni infection and hematological profile in an endemic foci in Western Burkina Faso. Journal of Parasitology Research. 2017;4(10).

[8] Eyayu T, Zeleke AJ, Seyoum M, Worku L. Basic coagulation profiles and platelet count among Schistosoma mansoni-infected adults attending Sanja Primary Hospital, Northwest Ethiopia. Research and Reports in Tropical Medicine. 2020;11:27.

[9] Dessie N, Lema W, Aemero M. Hematological and Biochemical Profile of Patients Infected with Schistosoma mansoni in Comparison with Apparently Healthy Individuals at Sanja Town, Northwest Ethiopia: A Cross-Sectional Study. Journal of tropical medicine. 2020;2020:4083252.

[10] Greenham R. Anaemia and Schistosoma haematobium infection in the North-Eastern Province of Kenya. Transactions of the Royal Society of Tropical Medicine and Hygiene. 1978;72(1):72-5.

[11] Wilkins H, Goll P, Moore P. Schistosoma haematobium infection and haemoglobin concentrations in a Gambian community. Annals of Tropical Medicine & Parasitology. 1985;79(2):159-61.

[12] Bhargava A, Jukes M, Lambo J, Kihamia C, Lorri W, Nokes C, et al. Anthelmintic treatment improves the hemoglobin and serum ferritin concentrations of Tanzanian schoolchildren. Food and nutrition bulletin. 2003;24(4):332-42.

[13] Mohammed EH, Eltayeb M, Ibrahim H. Haematological and biochemical morbidity of Schistosoma haematobium in school children in Sudan. Sultan qaboos university medical journal. 2006;6(2):59.

[14] Afrifa J, Gyedu D, Ofori Gyamerah E, Essien-Baidoo S, Mensah-Essilfie I. Haematological Profile and Intensity of Urogenital Schistosomiasis in Ghanaian Children. Journal of environmental and public health. 2017;2017:4248325.

[15] Dejon-Agobé JC, Adegnika AA, Grobusch MP. Haematological changes in Schistosoma haematobium infections in school children in Gabon. Infection. 2021;49:645–51.

[16] Sumbele I, Otia O, Francis L, Bopda O, Ebai C, Ning T, et al. Confounding influences of malnutrition and Plasmodium falciparum and Schistosoma haematobium infections on haematological parameters in school children in Muyuka, Cameroon. BMC infectious diseases. 2021;21(1):477.
